# Nurse Manager Empowerment and Nurses’ Engagement in Continuing Professional Development: A Parallel Mediation Analysis

**DOI:** 10.1155/jonm/4124590

**Published:** 2026-09-26

**Authors:** Naji Alqahtani, Batool N. AlQadoom

**Affiliations:** ^1^ Nursing Administration and Education Department, College of Nursing, King Saud University, Riyadh 11421, Saudi Arabia, ksu.edu.sa; ^2^ College of Nursing, King Saud University, Riyadh 11421, Saudi Arabia, ksu.edu.sa

**Keywords:** behavior, continuous professional development, empowerment, leadership, nurses, nursing, Saudi Arabia

## Abstract

**Background:**

Nurse manager empowerment is integral to creating supportive work environments that may foster nurses’ engagement in continuing professional development (CPD). Despite empowerment and CPD have been connected to positive professional outcome, limited research has explored the association between nurses’ perceived nurse manager empowerment and CPD engagement and the mechanisms that may contribute to this association.

**Aim:**

This study examines the association between nurses’ perceptions of nurse manager perceived empowerment and their CPD engagement in Saudi Arabia. It also assesses whether CPD motives, conditions, and perceived importance mediate this association.

**Methods:**

A quantitative, cross‐sectional, descriptive correlational design was adopted. Data were collected via online and paper‐based questionnaires from nurses working in the selected hospitals. Perceived empowerment and CPD were measured using the Nurse Manager Empowering Behavior Scale (NMEB‐SN) and the Questionnaire for Professional Development of Nurses (Q‐PDN). Data were analyzed using descriptive statistics, Pearson’s correlations, and multiple linear regression. Parallel mediation was tested using PROCESS Macro Version 5 (Model 4) with bootstrapping to examine the mediating roles of CPD motives, conditions, and perceived importance.

**Results:**

Nurses reported high perceived empowerment, strong CPD motivation, and high perceived importance of CPD. However, CPD condition scores were low, and overall CPD engagement was moderate. Perceived empowerment was positively correlated with CPD engagement (*r* = 0.448, *p* < 0.001). Among the proposed mediators, only perceived CPD importance partially mediated the relationship between perceived empowerment and CPD engagement.

**Conclusion:**

Nurse manager empowerment was positively associated with nurses’ CPD engagement and may support greater participation in CPD activities. Perceived CPD importance is a significant partial mediator of this association.

**Implications for Nursing Management:**

Nurse managers can foster work environments that support autonomy, recognition, and professional development. Enhancing empowerment may support nurses’ engagement in CPD.

## 1. Introduction

A supportive nursing work environment is essential, as it is associated with improved clinical practice and patient outcomes [1–3]. In nursing, empowerment—whether structural or psychological—has been linked to greater satisfaction with the quality of care, lower burnout, and stronger job retention [4–6]. Empowered nurses also tend to report better well‐being, higher professional engagement, greater autonomy, and more positive work‐related outcomes, all of which may contribute to improved patient care [3, 7, 8]. Nurse managers play an important role in fostering empowerment by offering support, access to learning opportunities, and greater autonomy in practice [7, 8].

Continuing professional development (CPD) is also central to nurses’ career development, as it supports safe and ethical practice, professional competence, clinical skills, and evidence‐based practice (EBP) [9, 10]. It helps nurses remain current in a changing healthcare environment and respond effectively to patient needs [9, 11]. As a lifelong process of active learning, CPD enables nurses to strengthen their practices and pursue career goals [9]. However, despite its well‐documented benefits, access barriers may limit participation and reduce its impact [10–12].

Nursing has evolved from a humanitarian service into a professional discipline that requires continuous education and lifelong learning. Accordingly, healthcare institutions must foster a culture of professional growth to sustain a motivated, skilled, and competent nursing workforce [9, 11]. Nursing leaders and healthcare managers should provide opportunities for professional development, while nurses also remain responsible for their own growth and learning [9, 11]. These efforts are critical because limited CPD opportunities may adversely affect nurse retention, quality of care, and patient safety [1–3, 13].

The current evidence has established that empowering leadership and supportive work environments are associated with positive nursing outcomes [4–8]. The CPD literature, on the other hand, has identified factors such as motivation, perceived value, and work conditions to influence nurses’ engagement in professional development [9, 10, 14, 15]. However, these literature works have been developed in parallel. Therefore, direct evidence linking nurses’ perceptions of nurse manger empowerment with actual CPD engagement remains limited, specifically in Saudi Arabia. Hence, the mechanisms that may contribute to this association are not well established in the literature. Empowerment studies have focused on the relation of autonomy, job satisfaction, and retention [4–8, 16–18]. CPD studies, however, have focused on barriers, facilitators, and motives [9, 10, 14, 15, 19]. Therefore, to the author knowledge, the indirect roles of CPD motives, CPD conditions, and perceived CPD importance in the association between perceived nurse manager empowerment and nurses’ CPD engagement have not been examined together in a parallel mediation model. By examining these associations, this study sought to clarify the mechanisms that may influence CPD engagement among nursing staff.

### 1.1. Theoretical Framework

**FIGURE 1:**
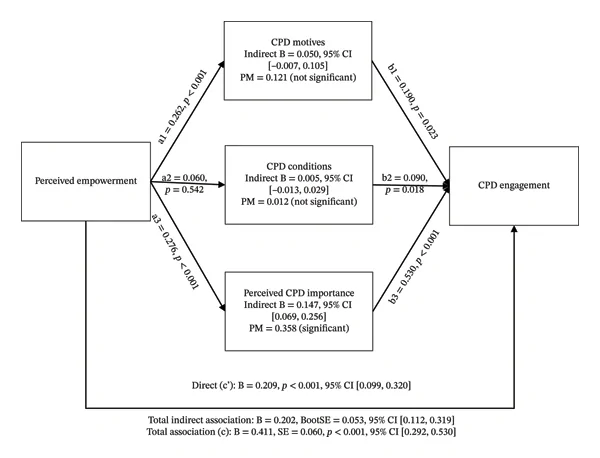
Conceptual framework and parallel mediation model. Note: Based on 5000 bootstrap samples, indirect coefficient is statistically significant when its 95% CI does not include zero. B = unstandardized coefficients; BootSE = bootstrap standard error; PM = proportion mediated; CPD = continuing professional development.

Self‐determination theory (SDT) was used to guide this study. The SDT explains how social and organizational environments are linked to motivation through meeting psychological needs such as autonomy, competence, and relatedness [20, 21]. Two subtheories, cognitive evaluation theory and organismic integration theory, were used further to explain the role of contextual support in strengthening intrinsic motivation and facilitate external motivated behavior [20, 21]. In the current study, nurse manager empowering behaviors were linked to organizational supports that may enhance nurses’ autonomy through empowering behaviors such as participation in decision‐making, recognition, competence, and supportive professional relationships [20–22]. These mechanisms support linking nurses’ perceptions of nurse manager empowerment with their CPD motives, perceived CPD conditions, and perceived CPD importance, which may be associated with higher CPD engagement [14]. Therefore, SDT supports the examination of CPD motives, perceived CPD conditions, and perceived CPD importance as parallel mediators in the association between perceived nurse manager empowerment CPD engagement (Figure 1).

## 2. Materials and Methods

### 2.1. Research Design

A descriptive correlational, cross‐sectional design was used to examine the associations among perceived nurse manager empowerment, CPD motives, CPD conditions, perceived CPD importance, and CPD engagement. The parallel mediation analysis was used to estimate the indirect associations among the study variables without assuming the causal effect.

### 2.2. Research Setting

The study was conducted in three hospitals in Riyadh, Saudi Arabia. The hospitals represent teaching, public, and private healthcare sectors, which vary in organizational context and possible CPD opportunities.

### 2.3. Population and Sample

The target population comprised registered staff nurses employed at one of the selected hospitals. Participants were recruited using convenience sampling. Eligible nurses who could be reached at the organizations’ official communication system or during department visit, provided direct patient care, had worked at their current institution for at least 6 months, and voluntarily agreed to participate. The 6‐month period was added to inclusion criteria to ensure sufficient exposure to the workplace environment and the current behavior of the current nurse manager’s behavior. Nurses in managerial or supervisory roles, including head nurses or charge nurses, were excluded because the study focused on staff nurses who are the recipients of nurse manager empowering behaviors.

#### 2.3.1. Sample Size Calculation

G∗Power software Version 3.1 was used to calculate the minimum required sample size for the statistical analysis. At a significance level of 0.05, effect size of 0.15, power of 0.9, and 13 independent variables, the minimum sample size required to run a multiple regression analysis was 162 participants. For the mediation modeling, the sample size provides adequate statistical power. Of the 600 invited nurses, 194 nurses completed the questionnaires, corresponding to a response rate of 32.2%.

### 2.4. Measurements

Data were collected using structured questionnaires. Four instruments were used in this study.

#### 2.4.1. Demographic Measures

Demographic variables included age, sex, nationality, educational level, years of experience, years working with the current manager, hospital type, shift type, and previous CPD participation.

#### 2.4.2. Perceived Empowerment

Perceived empowerment was measured using the Nurse Manager Empowering Behavior Scale (NMEB‐SN), developed by Sasaki et al. [22]. The scale assesses staff nurses’ perceptions of nurse managers’ empowering behaviors. It contains 15 items and 5 subscales: providing meaning to work, supporting autonomy to enhance self‐confidence, helping staff overcome work‐related obstacles, recognizing work, and respecting staff members. Items are rated on a 5‐point Likert scale, with higher scores indicating greater perceived empowerment and more empowering managerial behaviors. The instrument has demonstrated strong psychometric properties, including internal consistency, construct validity, criterion validity, and test–retest reliability [22]. Reported Cronbach’s alpha coefficients were greater than 0.90 for each subscale and 0.98 for the full scale [22].

#### 2.4.3. CPD

CPD was measured using a Questionnaire for Professional Development of Nurses (Q‐PDN) developed by Brekelmans et al. [14]. The Q‐PDN is a multidimensional 54‐item instrument that assesses nurses’ perceptions of and engagement in CPD. It includes four dimensions: CPD motives (12 items), CPD conditions (12 items), perceived importance of CPD (15 items), and CPD engagement (15 items). Items are rated on a 5‐point Likert scale, with higher scores indicating stronger CPD motives, more favorable CPD conditions, greater perceived importance of CPD, and higher engagement in CPD activities, respectively. Reliability analysis showed satisfactory to good Cronbach’s alpha coefficients across all factors, ranging from 0.70 to 0.89 [14].

### 2.5. Ethical Considerations

Ethical approval was obtained from the King Saud University Ethics Committee (Approval No. KSU‐HE‐25755) and the Saudi Ministry of Health (Approval No. 25‐519E) before data collection. Participation was voluntary, and eligible participants received full information about the study’s purpose, procedures, their rights, and they could withdraw without any penalty before providing informed consent. Electronic and written informed consent was obtained from all participants prior to their participation. Confidentiality and privacy were maintained by anonymizing responses and collecting no personal identifiers. All data were securely stored in a secured device in a locked room at the university campus to ensure data protection. The data will be destroyed after 5 years of completing the study. Only the research team has access to the study data.

### 2.6. Data Collection Procedure

After ethical approval was obtained, data were collected using self‐administered questionnaires in both online and paper‐based formats to maximize participation and accommodate participant preferences. The researcher distributed the online questionnaire to nurses’ official email addresses through a centralized communication system. The researcher also visited nursing departments of each hospital to collect data using paper‐ and ‐pencil questionnaires. Only eligible participants were invited to participate in the study.

### 2.7. Data Analysis Plan

Data were analyzed using SPSS Version 31. Descriptive statistics and measures of central tendency were calculated. Pearson’s correlation coefficients were used to examine associations among variables. Multiple linear regression was conducted to assess the associations of empowerment, CPD motives, CPD conditions, and perceived CPD importance on CPD engagement while controlling for demographic variables. The parallel mediating indirect association of CPD motives, CPD conditions, and perceived importance of CPD on the relationship between empowerment and CPD engagement were examined using PROCESS Macro v.5, Model 4, with bootstrapping, following the protocol described by Hayes [23].

## 3. Results

### 3.1. Demographic Characteristics

The sample comprised 194 nurses. The mean age was 32.3 (standard deviation [SD] = 7.51, range: 22–55 years; Table 1). Most participants were female (84.0%), expatriate nurses (85.6%), and held a bachelor’s degree (77.8%). The mean years of experience was 7.58 (SD = 5.84, range: 0.5–25 years). Most participants worked 12‐h shifts (77.3%) and had previously participated in CPD (66.5%). More than 41% were employed in public hospitals.

**TABLE 1:** Demographic characteristics of participants (*N* = 194).

Variable (range)	*n* (%) or mean ± SD
Age (22–55 years)	32.3 ± 7.51
Sex	
Female	163 (84.0)
Male	31 (16.0)
Nationality	
Saudi	28 (14.4)
Non‐Saudi	166 (85.6)
Level of education	
Diploma	45 (23.2)
Bachelor	149 (77.8)
Years of experience (0.5–25)	7.58 ± 5.84
Years of working with the current manager (0–16)	2.51 ± 2.87
Hospital type	
Teaching	62 (32.0)
Public	81 (41.8)
Private	51 (26.3)
Shift type	
8‐h	44 (22.7)
12‐h	150 (77.3)
Previous CPD participation	
Yes	129 (66.5)
No	65 (33.5)

### 3.2. Descriptive Statistics

The mean total score for perceived empowerment was high (4.13, SD = 0.98; Table 2). Among the empowerment subscales, supporting autonomy had the highest mean score (4.19, SD = 0.96), followed by providing meaning to work (mean = 4.15, SD = 1.06), supporting staff to overcome obstacles (mean = 4.12, SD = 1.04), recognizing work (mean = 4.10, SD = 1.04), and respecting staff members (mean = 4.10, SD = 1.86).

**TABLE 2:** Descriptive statistics.

Variable (possible range)	Range	Mean (SD)
Perceived empowerment (total average) (1–5)	1–5	4.13 (0.98)
Providing meaning to work (1–5)	1–5	4.15 (1.06)
Supporting autonomy (1–5)	1–5	4.19 (0.96)
Providing support to overcome obstacles (1–5)	1–5	4.12 (1.04)
Recognizing work (1–5)	1–5	4.10 (1.04)
Respecting as a staff member (1–5)	1–5	4.10 (1.86)
CPD motivation (total average) (1–5)	1–5	4.25 (0.80)
CPD importance (total average) (1–5)	1.48–5	4.17 (0.65)
CPD conditions (total average) (1–5)	1–5	2.89 (1.33)
CPD engagement total average) (1–5)	1.26–5	3.60 (0.91)

For CPD‐related variables, participants reported high CPD motivation (mean = 4.25, SD = 0.80) and high perceived importance of CPD (mean = 4.17, SD = 0.65). However, CPD conditions had a lower mean score (mean = 2.89, SD = 1.33), and CPD engagement was moderate (mean = 3.6, SD = 0.91).

### 3.3. Relationship Analysis

Perceived empowerment was positively and significantly correlated with CPD conditions (*r* = 0.323, *p* < 0.001; Table 3), perceived CPD importance (*r* = 0.416, *p* < 0.001), and actual CPD engagement (*r* = 0.448, *p* < 0.001). CPD motivation was strongly correlated with perceived CPD importance (*r* = 0.655, *p* < 0.001) and moderately correlated with CPD engagement (*r* = 0.505, *p* < 0.001). Perceived CPD importance was also strongly and positively correlated with CPD engagement (*r* = 0.599, *p* < 0.001). In contrast, CPD conditions showed a weak but significant correlation with CPD engagement (*r* = 0.179, *p* < 0.05).

**TABLE 3:** Correlation matrix.

Variable	1	2	3	4	5
1 Perceived empowerment	1				
2 CPD motivation	0.323^∗∗∗^	1			
3 CPD importance	0.416^∗∗∗^	0.655^∗∗∗^	1		
4 CPD conditions	0.039	0.078	0.063	1	
5 CPD engagement	0.448^∗∗∗^	0.505^∗∗∗^	0.599^∗∗∗^	0.179^∗^	1

^∗^ *p* < 0.05.

^∗∗∗^ *p* < 0.001.

Multiple regression analysis (Table 4) revealed that the overall model was statistically significant and explained 50.2% of the variance in CPD engagement (*R*2 = 0.502, *p* < 0.001). Among the demographic variables, only nationality was significantly associated with CPD engagement (*β* = 0.168, *p* < 0.05). Among the main study variables, perceived empowerment (*β* = −0.201, *p* < 0.01), CPD motivation (*β* = 0.173, *p* < 0.05), and perceived CPD importance (*β* = 0.412, *p* < 0.001) were positively associated with CPD engagement.

**TABLE 4:** Multiple linear regression analysis of CPD engagement.

Independent variables	CPD engagement			
	B^a^	β^b^	t	Model summary
Age	0.004	0.033	0.307	R^2^ = 0.502 *F* (14, 175) = 5.606 *p* = < 0.001^∗∗∗^
Sex (female)^†^	−0.077	−0.031	−0.517	
Nationality (non‐Saudi)^†^	**0.431** ^∗^	**0.168** ^∗^	**2.579** ^∗^	
Level of education (bachelor)^†^	−0.037	−0.017	−0.236	
Years of experience	−0.013	−0.087	−0.769	
Years of working with the current manager	−0.040	−0.126	−1.921	
Hospital type: Teaching	0.120	0.062	0.816	
Hospital type: Private	0.036	0.017	0.175	
Shift type (12‐h)^†^	−0.048	−0.022	−0.344	
Previous CPD participation	−0.111	−0.057	−0.950	
Perceived empowerment	**0.185** ^∗∗^	**0.201** ^∗∗^	**2.998** ^∗∗^	
CPD motivation	**0.195** ^∗^	**0.173** ^∗^	**2.313** ^∗^	
CPD importance	**0.571** ^∗∗∗^	**0.412** ^∗∗∗^	**5.292** ^∗∗∗^	
CPD conditions	0.069	0.101	1.821	

^†^Reference group.

^a^B coefficient.

^b^Beta standardized coefficient.

^∗^ *p* < 0.05.

^∗∗^ *p* < 0.01.

^∗∗∗^ *p* < 0.001.

### 3.4. Parallel Mediation Analysis

Perceived empowerment showed a significant direct association with CPD engagement (*B* = 0.209, *p* < 0.001; Table 5). It also had significant association with CPD motives (*B* = 0.262, *p* < 0.001) and perceived CPD importance (*B* = 0.276, *p* < 0.001) but not with CPD conditions (*B* = 0.060, *p* = 0.542). Additionally, CPD motives (*B* = 0.190, *p* = 0.023), CPD conditions did not (*B* = 0.090, *p* = 0.18), and perceived CPD importance (*B* = 0.530, *p* < 0.001) were each significantly associated with CPD engagement. Bootstrap analysis showed that only perceived CPD importance partially mediated the association between perceived empowerment and CPD engagement (*B* = 0.147; 95% CI: [0.069–0.256]). The proportion mediated (PM) was 0.358, indicating that 35.8% of the total effect of perceived empowerment on CPD engagement was explained by the indirect association through perceived CPD importance.

**TABLE 5:** Parallel mediation association of CPD motives, importance, and condition by bootstrapping.

						95% CI		P_M_
Effect	Variable	B	SE	t	*p*	LLCI	ULCI	
Direct	Empowerment ⟶ CPD engagement (c’)	0.209	0.056	3.737	0.000	0.099	0.320	
Indirect	Empowerment ⟶ Motives (a1)	0.262	0.056	4.643	0.000	0.151	0.373	
Indirect	Motives ⟶ CPD engagement (b1)	0.190	0.082	2.299	0.023	0.027	0.354	
Indirect	Empowerment ⟶ Motives ⟶ CPD engagement (ab1)	0.050	0.028			−0.007	0.105	0.121
Indirect	Empowerment ⟶ Condition (a2)	0.060	0.098	0.610	0.542	−0.134	0.254	
Indirect	Condition ⟶ CPD engagement (b2)	0.090	0.038	2.386	0.018	0.016	0.165	
Indirect	Empowerment ⟶ Condition ⟶ CPD engagement (ab2)	0.005	0.010			−0.013	0.029	0.012
Indirect	Empowerment ⟶ Importance (a3)	0.276	0.044	6.273	0.000	0.189	0.363	
Indirect	Importance ⟶ CPD engagement (b3)	0.530	0.106	5.003	0.000	0.321	0.739	
Indirect	Empowerment ⟶ Importance ⟶ CPD engagement (ab3)	0.147	0.048			0.069	0.256	0.358
Total indirect	Total indirect effect (ab1 + ab2 + ab3)	0.202	0.053			0.112	0.319	0.491
Total effect	(c’ + ab1 + ab2 + ab3)	0.411	0.060	6.835	0.000	0.292	0.530	

*Note:* 5000 bootstrapping re‐extracted. B = B coefficient; LLCI = lower limit of B in 95% CI; ULCI = upper limit of B in 95% CI; PM = proportion mediated (ratio of the indirect effect to the total effect).

Abbreviations: CI, confidence interval; SE, standard error.

## 4. Discussion

This study examined the relationship between perceived empowerment and CPD engagement among nurses in Saudi Arabia and assessed the parallel mediating roles of CPD motives, conditions, and perceived importance. Overall, nurses reported high levels of perceived nurse manager empowerment, strong CPD motivation, and high perceived importance of CPD. However, CPD condition scores were low, and CPD engagement was moderate. These findings suggest that although nurses value CPD and are motivated to participate, workplace conditions may not adequately support their engagement. CPD conditions refer to organizational, environmental, and personal conditions a nurse need to successfully engage in CPD [14]. This is consistent with previous research showing that, despite recognizing the importance of CPD, nurses’ participation may be constrained by heavy workload, limited time, scheduling constraints, staff shortages, restricted access to learning opportunities, and insufficient financial and organizational support [9, 10, 14, 15]. Similar challenges have been reported in Saudi Arabia, highlighting the need for more accessible professional development opportunities that align with nurses’ needs [19]. The findings suggest that valuing and being motivated to participate in CPD activities may not be sufficient when organizational conditions do not facilitate engagement.

Perceived nurse–manager empowerment was positively associated with CPD engagement, underscoring the important role of nurse managers in supporting nurses’ participation in professional development. Empowering managerial behaviors—such as providing meaning to work, supporting autonomy, helping overcome obstacles, recognizing contributions, and showing respect—may foster a positive work environment that encourages engagement in CPD [22]. This finding is consistent with international and Saudi studies linking empowerment to favorable nurse outcomes, including job satisfaction, work engagement, intention to stay, and organizational commitment [4, 5, 7, 16–18]. Thus, perceived empowerment appears to be important not only for positive nurse outcomes but also for CPD engagement. The finding that Saudi nurses reported higher CPD engagement than non‐Saudi nurses may reflect ongoing national efforts to increase the number of Saudi nurses through Vision 2030 and the Health Sector Transformation Program, which have expanded opportunities for nursing education and professional development and elevated the status of the nursing profession in Saudi Arabia [24, 25].

Regression analysis showed that perceived empowerment, CPD motivation, and perceived CPD importance were significant predictors of nurses’ CPD engagement. Perceived CPD importance had the strongest association with engagement, indicating that nurses’ beliefs about the value of CPD were more strongly associated with CPD engagement than motivational factors alone in the model. Previous studies similarly indicate that nurses value CPD in their clinical practice and professional development [9, 10]. These findings suggest that simply offering professional development opportunities may not be sufficient to increase engagement; nurses must also perceive CPD as relevant and beneficial to their clinical practice. Additionally, nationality was associated with CPD engagement, with Saudi nurses reporting higher engagement than non‐Saudi nurses.

Mediation analysis showed that perceived CPD importance was the only indirect significant mediator of the association between perceived empowerment and CPD engagement. Empowering nurse manger behaviors is associated with strong perceptions of CPD importance. Such perceptions were associated with greater CPD engagement. This finding suggests that nurse managers may enhance CPD engagement by strengthening nurses’ perceptions of its importance. This finding is consistent with evidence that managerial support and autonomy could foster professional outcomes [7, 8, 22], and nurses’ perceived CPD importance is important for CPD engagement and participation [9, 10, 14]. To our knowledge, this is the first study to examine perceived CPD importance as a mediator between perceived empowerment and CPD engagement, while previous research has examined empowerment and CPD‐related factors independently. On the other hand, CPD motivation and CPD conditions did not significantly mediate this association in the parallel model. This may indicate that motivation alone is insufficient to increase CPD engagement, as nurses may remain unable to participate due to competing responsibilities. Similarly, the nonsignificant mediating association of CPD conditions may reflect the relatively low CPD condition scores observed in this study. Previous studies have similarly shown that improving work‐related factors and reducing barriers to CPD access are important for maximizing nurses’ participation in CPD [15, 19]. This suggests that broader organizational and structural conditions, beyond individual nurse manager behaviors, may be relevant to nurses CPD engagement.

### 4.1. Implications for Nursing Management

These findings suggest that nurse managers may benefit from adopting empowering behaviors that support autonomy, recognize nurses’ contributions, and help them overcome barriers to professional development. Healthcare leaders should also consider improving CPD conditions by providing protected time, funding, and accessible learning opportunities. Moreover, nurse managers may enhance engagement by clearly communicating the value of CPD and linking CPD activities to nurses’ clinical needs, such as patient safety, EBP, quality improvement, and career development. Organizations may also benefit from leadership development programs that prepare nurse managers to support staff nurses’ professional development and lifelong learning.

### 4.2. Limitations of the Study

This study has several limitations that should be considered when interpreting the results. First, the cross‐sectional design precludes conclusions about causality between perceived nurse manager empowerment and nurses’ CPD engagement. Although PROCESS can estimate the indirect effect in the cross‐sectional design, these estimates should not be interpreted as causal mediation. Therefore, longitudinal or experimental studies are needed to establish the causal effect. Second, the study was conducted only at large institutions in Riyadh, which may limit the transferability of the findings to other healthcare settings with different organizational structures. Third, convenience sampling may have introduced sampling bias. Fourth, the use of self‐report questionnaires may have introduced response bias, including social desirability and overestimation. Future research should use longitudinal or mixed‐methods designs to examine these relationships more comprehensively and explore nurses’ experiences in greater depth. Replicating the study across different healthcare institutions, geographic regions, and specialties in Saudi Arabia would help strengthen the findings’ broader applicability.

## 5. Conclusions

This study found that perceived nurse manager empowerment was positively associated with nurses’ CPD engagement. Among the three mediators, only perceived CPD importance showed was a significant indirect association of this relationship. Although nurses reported high CPD motivation and perceived importance, CPD conditions were low, which underscore the need for stronger organizational support with empowering leadership behavior. Creating supportive and empowering work environments, communicating the relevance of CPD, and reducing barriers to CPD participation may help strengthen nurses’ engagement in CPD within professional nursing practice.

## Funding

This work was supported by the Ongoing Research Funding Program of King Saud University [ORF‐2026‐856].

## Conflicts of Interest

The authors declare no conflicts of interest.

## Data Availability

The data that support the findings of this study are available from the corresponding author upon reasonable request.
